# Abnormal Histopathological Expression of Klotho, Ferroptosis, and Circadian Clock Regulators in Pancreatic Ductal Adenocarcinoma: Prognostic Implications and Correlation Analyses

**DOI:** 10.3390/biom14080947

**Published:** 2024-08-05

**Authors:** Cielo García-Montero, Oscar Fraile-Martinez, David Cobo-Prieto, Diego De Leon-Oliva, Diego Liviu Boaru, Patricia De Castro-Martinez, Leonel Pekarek, Raquel Gragera, Mauricio Hernández-Fernández, Luis G. Guijarro, María Del Val Toledo-Lobo, Laura López-González, Raul Díaz-Pedrero, Jorge Monserrat, Melchor Álvarez-Mon, Miguel A. Saez, Miguel A. Ortega

**Affiliations:** 1 Department of Medicine and Medical Specialities (CIBEREHD), Faculty of Medicine and Health Sciences, University of Alcalá, 28801 Alcala de Henares, Spain; cielo.gmontero@gmail.com (C.G.-M.); oscarfra.7@gmail.com (O.F.-M.); dcobpri@gmail.com (D.C.-P.); diegodleonoliva01@gmail.com (D.D.L.-O.); diego.boaru@edu.uah.es (D.L.B.); patriciadecastro1999@gmail.com (P.D.C.-M.); leonel.pekarek@gmail.com (L.P.); raquel.gragera@uah.es (R.G.); jorge.monserrat@uah.es (J.M.); mademons@gmail.com (M.Á.-M.); msaega1@oc.mde.es (M.A.S.); 2 Ramón y Cajal Institute of Sanitary Research (IRYCIS), 28034 Madrid, Spain; luis.gonzalez@uah.es (L.G.G.); mval.toledo@uah.es (M.D.V.T.-L.); laura.lgonzalez@uah.es (L.L.-G.); raul.diazp@uah.es (R.D.-P.); 3 Immune System Diseases-Rheumatology Service, Central University Hospital of Defence-UAH Madrid, 28801 Alcala de Henares, Spain; 4 Department of Surgery, Medical and Social Sciences, Faculty of Medicine and Health Sciences, University of Alcalá, 28801 Alcala de Henares, Spain; lmauricio.hernandez@uah.es; 5 Unit of Biochemistry and Molecular Biology, Department of System Biology (CIBEREHD), University of Alcalá, 28801 Alcala de Henares, Spain; 6 Department of Biomedicine and Biotechnology, Faculty of Medicine and Health Sciences, University of Alcalá, 28801 Alcala de Henares, Spain; 7 Immune System Diseases-Rheumatology and Internal Medicine Service, University Hospital Prince of Asturias, Networking Research Center on for Liver and Digestive Diseases (CIBEREHD), 28806 Alcala de Henares, Spain; 8 Pathological Anatomy Service, University Hospital Gómez-Ulla, 28806 Alcala de Henares, Spain

**Keywords:** pancreatic ductal adenocarcinoma (PDAC), ferroptosis markers, TFRC, circadian regulators, CLOCK, KLOTHO, prognosis

## Abstract

Pancreatic ductal adenocarcinoma (PDAC) is an extremely lethal tumor with increasing incidence, presenting numerous clinical challenges. The histopathological examination of novel, unexplored biomarkers offers a promising avenue for research, with significant translational potential for improving patient outcomes. In this study, we evaluated the prognostic significance of ferroptosis markers (TFRC, ALOX-5, ACSL-4, and GPX-4), circadian clock regulators (CLOCK, BMAL1, PER1, PER2), and KLOTHO in a retrospective cohort of 41 patients deceased by PDAC. Immunohistochemical techniques (IHC) and multiple statistical analyses (Kaplan–Meier curves, correlograms, and multinomial linear regression models) were performed. Our findings reveal that ferroptosis markers are directly associated with PDAC mortality, while circadian regulators and KLOTHO are inversely associated. Notably, TFRC emerged as the strongest risk marker associated with mortality (HR = 35.905), whereas CLOCK was identified as the most significant protective marker (HR = 0.01832). Correlation analyses indicate that ferroptosis markers are positively correlated with each other, as are circadian regulators, which also positively correlate with KLOTHO expression. In contrast, KLOTHO and circadian regulators exhibit inverse correlations with ferroptosis markers. Among the clinical variables examined, only the presence of chronic pathologies showed an association with the expression patterns of several proteins studied. These findings underscore the complexity of PDAC pathogenesis and highlight the need for further research into the specific molecular mechanisms driving disease progression.

## 1. Introduction

Pancreatic cancer is a highly lethal cancer currently representing the seventh leading cause of cancer deaths globally [1]. It is projected that the incidence of pancreatic cancer will present an average annual growth of 1.1%, becoming the second leading cause of cancer death in some regions [2]. Pancreatic ductal adenocarcinoma (PDAC) accounts for more than 90% of all pancreatic malignancies [3]. PDAC is often diagnosed at advanced stages, and despite the irruption of new surgical techniques and medical procedures, only modest improvements in clinical routine have been achieved [4]. Scientific literature agrees that the identification of serum and histopathological biomarkers represents a pivotal point current and future of study in PDAC [5]. Despite some biomarkers being currently used in the clinical routine of PDAC, the potential of deepening on novel histopathological indicators is growingly being supported by compelling evidence [6]. In this sense, the discovery and description of unexplored histopathological markers has provided valuable information on the prognosis of patients with PDAC, also allowing the opening of therapeutic lines and other potential translational approaches [7,8,9,10,11,12].

Ferroptosis is as an iron-dependent form of regulated cell death caused by persistent lipid peroxidation and subsequent membrane damage consequent of oxidative stress phenomena [13]. Ferroptosis is a process with demonstrated carcinogenic and antitumoral actions depending on the context and type of tumor [14]. A growing body of literature supports the prognostic relevance of ferroptosis biomarkers in different types of cancer [15,16,17]. In the event of pancreatic cancer, preliminary evidence suggests that this cellular process may play an important role in the initiation and development of these types of tumors, also offering a potential prognostic role yet to be explored [18,19,20]. The transferrin receptor (TFRC); the acyl-CoA synthetase long-chain family member 4 (ACSL-4); arachidonate lipoxygenase-5 (ALOX-5); and glutathione peroxidase 4 (GPX4) are four well-established biomarkers of ferroptosis [21,22]. The relevance of these ferroptosis-related molecules in pancreatic cancer have been proven by past works [23,24,25,26]; however, the prognostic value derived from the histopathological study of these molecules remains to be fully covered.

On the other hand, we also aimed to explore additional molecular pathways which could also be used as prognostic markers in PDAC. In this sense, circadian disruption seems to be a major mechanism involved in the initiation and development of multiple types of cancer [27]. Circadian rhythms are controlled by a set of regulatory molecules like the protein CLOCK (circadian locomotor output cycles kaput), brain and muscle ARNT-like protein 1 (Bmal1), PER1 and PER2, all of which seems to be dysregulated in cancer [28,29]. A growing body of evidence is reporting that the circadian clock is dysregulated in PDAC, leading to different mechanisms of carcinogenesis, tumor growth, metastasis, and therapy resistance, making them ideal to act as potential biomarkers [30,31]. However, despite some preliminary evidence, the prognostic value of CLOCK, Bmal1, PER1, and PER2 in PDAC remains to be fully elucidated.

In a similar way, the protein KLOTHO is a molecule initially described as an antiaging molecule with multiple activities dysregulated in a set of diseases like cancer [32]. Downregulation of Klotho was found in several cancers, such as pancreatic cancer and other type of tumors [33]. The prognostic role of KLOTHO in cancer is starting to be supported by a growing number of studies [34], and some preliminary findings reported in vivo and in silico suggest that this protein might act as a tumor suppressor with potential prognostic implications [35]. However, direct histopathological observations and deepening on translational implications related to the dysregulation of this protein are still warranted.

In this sense, the objectives of the present work are to explore, by immunohistochemistry (IHC), protein expression of ferroptosis and chronobiological markers, together with KLOTHO in PDAC tissue obtained from 41 patients deceased by this condition and correlate their values with mortality. Additionally, another objective was to correlate the expression level of the different molecules included in our study between them. Finally, an association between different medical variables and the protein expression of these components will be performed in order to predict or gain further insights into the relationship between clinical and biological factors.

## 2. Patients and Methods

### 2.1. Study Characteristics and Sample Collection

This research used paraffin-embedded pancreatic tissue sections from 41 patients with ductal adenocarcinoma who underwent pancreatoduodenectomy surgery (curative resection). The patients were tracked for a period of 60 months, with diagnoses following previously established guidelines [36]. The study was designed as a retrospective, analytical, observational cohort study with extended follow-up. Reviews were conducted on the paraffin blocks, along with comprehensive clinical information and follow-up data for each patient.

The study adhered to Good Clinical Practice guidelines, the principles of the latest Declaration of Helsinki (2013), and the Convention of Oviedo (1997). It was conducted following the ethical principles of autonomy, beneficence, non-maleficence, and distributive justice. Data collection complied with the latest data protection laws, including Regulation (EU) 2016/679 and Organic Law 3/5 December 2018, which safeguards personal data and ensures digital rights.

### 2.2. Histopathological Analysis (Immunohistochemistry) and Evaluation

Pancreatic tissue samples preserved in paraffin were analyzed using immunohistochemical techniques. The protocol specifications (Table 1) included details on the antibody retrieval step. Antigen/antibody interactions were detected using the avidin–biotin (ABC) complex method combined with avidin–peroxidase, following established procedures [37]. The samples were incubated overnight at 4 °C with a 3% BSA blocker (Catalog #37525; Thermo Fisher Scientific, Inc., Waltham, MA, USA) and PBS after a 90-min incubation with the primary antibody. Subsequently, the samples were incubated for 90 min at room temperature with a biotin-conjugated secondary antibody diluted in PBS (rabbit IgG, diluted 1/300 (RG-96; Sigma-Aldrich, St. Louis, MO, USA), goat IgG, diluted 1/100 (GT-34/B3148; Sigma-Aldrich), and mouse IgG, diluted 1/300 (F2012/045K6072; Sigma-Aldrich)). ExtrAvidin^®^-Peroxidase (Sigma-Aldrich; Merck KGaA, Darmstadt, Germany), an avidin and peroxidase conjugate, was applied for 60 min at room temperature (1:200 with PBS). A Chromogenic Diaminobenzidine (DAB) Substrate Kit (cat. no. SK-4100; Maravai LifeSciences, San Diego, CA, USA) was then employed to visualize protein expressions. The kit, prepared immediately before use, contained five milliliters of distilled water, two drops of buffer, four drops of DAB, and two drops of hydrogen peroxide. Brown staining indicated protein presence, as the chromogenic peroxidase substrate produced a signal after 15 min at room temperature. For each protein, sections of the same tissue served as negative controls, with PBS used as the blocking solution instead of the primary antibody. Carazzi hematoxylin was used to counterstain each tissue section for 15 min at room temperature.

A Zeiss Axiophot light microscope (Carl Zeiss, Oberkochen, Germany) equipped with an AxioCam HRc digital camera (Carl Zeiss, Oberkochen, Germany) was used to examine the tissue slices. Given the importance of the proteins being studied, histological assessment was performed based on the intensity of immunohistochemistry staining using the IRS Score method. Histological samples from patients with pancreatic cancer were categorized as low/medium (1/2), high (3), and negative expression (0) [7,38]. In each of the five sections, seven randomly selected microscopy fields were evaluated for each patient group.

Patients were classified as positive when the average proportion of labeled sample was greater than or equal to 5% of the total sample. This was achieved by calculating the overall percentage of marked tissue in each microscope field to produce an average for the study sample, as described in previous studies [9]. Two histopathologists independently observed and quantified the samples.

### 2.3. Statistical Analysis

The software employed was Rstudio 2023.12.1 built 402.

(a) **Descriptive**

Demographic and carcinogenic markers were included in the initial descriptive analysis, where continuous numerical variables were presented as the median and interquartile range (IQR) and categorical were expressed as frequency (*n*, %).

(b) **Survival Analysis**

First, we addressed the median survival times and IQR corresponding to every level of expression for each protein marker. Then, we initiated the Kaplan–Meier curves to denote the survival progression associated with each protein marker. With log-rank test, we tested the statistically significant differences in survival between the groups defined by each marker’s expression levels. Consecutively, with the Cox proportional hazards model, we assessed if the risk of the event (death) changes with each protein expression calculating hazard ratios (HRs), their confidence levels (with fidelity of 95%), and values of the models.

(c) **Correlation Analysis**

In another step, we performed a simple correlation analysis to establish the possible relations of collinearity among the protein markers. The pairwise heatmap matrix of correlation was given with non-parametric Spearman coefficients. We exposed in cyan color the positive values (that is, directly related variables) and in magenta the negative ones (meaning inversely related variables). We made the correction of *p* values associated to Spearman coefficients through False Discovery Rate (FDR) correction to diminish the bias.

(d) **Fisher’s Exact Test and Multinomial Logistic Regression (MLR)**

In order to model the probability of each level of protein expression as a function of the predictor variables, which are categorical, we accomplished Multinomial Logistic Regression (MLR) models. To justify the models to adjust, we first applied Fisher’s Exact Test to see which variables had relevance in the protein markers expression, as well as the combinations of those variables.

In a second step, now that we conducted several Fisher’s Exact Tests, and we have limited sample size (*n*= 41), we applied a FDR correction with the Benjamini–Hochberg method to determine which results were truly significant and not a result of random chance.

Through the MLR models, we pursued to model the relationship between categorical dependent variables with more than two levels and predictor outcomes. On one hand, we set as categorical outcomes the protein expression levels considered as negative, low/medium, and high of TFRC, ACSL-4, ALOX-5, GPX4, Bmal 1, CLOCK, PER1, PER2, and KLOTHO. On the other hand, we set as predictor variables sex, smoking habit, drinking habit, obesity, type 2 diabetes + obesity (diabesity), chronic pathologies, prior malignancies, and stage IV condition in the moment of diagnosis). The package *nnet* will calculate coefficients representing the log odds of being in a specific category, and then the results are expressed in odds ratios, which are obtained with the function of exponentiation applied to those coefficients.

(e) **Kruskal–Wallis Test and Ordinal Logistic Regression (OLR) models**

Additionally, we assessed the possible relationship between the levels of each protein expression marker and the numerical clinical variables of age, and the carcinogenic measurable parameters commonly addressed in oncology clinics (Ca-19.9, CEA, and AFP). Eventually, we performed the Ordinal Logistic Regression (OLR) models in order to justify if there is a probability of transitioning from one group of expression to the consecutive one for each protein marker according to the predictive numerical parameters. We calculated the coefficients OLR and exponentiated them to express the ODDs ratios and also the *p* values, for those variables that resulted as statistically significant in the previous step of the Kruskal–Wallis test.

## 3. Results

### 3.1. Clinical and Sociodemographic Characteristics of the Studied Sample

The main clinical and sociodemographic characteristics of the patients in our study are summarized in Table 2. Our cohort was composed of 41 patients, including 27 men (65.85%) and 14 women (34.15%), with a median age of 72.00 years (range: 45.00–88.00 years). Of the patients, 43.9% were smokers, 26.83% had a history of alcohol consumption, 4.88% were obese, 55.56% had obesity and type 2 diabetes (diabesity), 9.76% had chronic illnesses, and 26.83% had previously been diagnosed with malignant neoplasms.

In parallel, 13 patients were diagnosed with stage IV tumors, while the other 28 patients had tumors classified as stage < IV (Table 3). The median levels of AFP, CEA, and CA19-9 in these patients were 2.32 [1.46–4.39] ng/mL, 5.43 [2.71–11.31] ng/mL, and 102.10 [44.91–805.00] U/mL, respectively. The average survival time for those with pancreatic cancer was 8.00 [2.98–13.02] months.

### 3.2. Immunohistochemistry and Kaplan–Meier Analysis

#### 3.2.1. Expression Levels of Ferroptosis, Circadian Regulation, and Antiaging Markers in PDAC Patients

Firstly, we aimed to explore protein expression for the markers of ferroptosis, circadian regulation and KLOTHO by IHC. We divided our patients into high (IRS score = 2), low/medium (IRS score = 1), and negative expression (IRS score = 0). Regarding ferroptosis markers, we observed that 31 patients with PDAC presented high expression of TFRC and ALOX-5 (75.61%), whereas only 6 (14.63%) exhibited lower/medium expression for both markers and 4 (9,76%) showed negative expression. For ACSL-4, 29 (70.73%) showed high expression of this protein, 11 (26.83%) low/medium, and 1 (2.44%) negative expression. For GPX4, 27 patients showed high expression of this protein (65.85%), 10 (24.39%) low/medium, and 4 (9,76%) negative expression.

For circadian clock markers, 5 patients showed high expression of Bmal1 (12.2%), 10 low/medium (24.39%), and 26 (63.41%) negative expression. For CLOCK, we observed that only 7 patients showed high expression of this marker (17.08%), 8 low/medium (19.51), and 26 negative expression (63.41%). Regarding PER1, we report high expression of this component in 9 patients (21.95%), 10 for low/medium (24.39%), and 22 for negative expression (53.66%). For PER2, we identified high expression levels in 6 subjects (14.63%), low/medium in 13 (31.71%), and negative expression in 22 (53.66%).

Finally, for KLOTHO, 5 individuals presented high expression levels (12.2%), 11 low/medium expression (26.82%), and 25 negative expression (60.98%).

Overall, we summarize in Table 4 the median survival time with the IQR as well as the number of patients with negative, low/medium, and high expression for each marker.

#### 3.2.2. Survival Analysis According to Protein Expression of Ferroptosis, Circadian, and Antiaging Markers in PDAC Patients

For analyzing patient survival according to the expression level of each protein, we performed Kaplan–Meier curves for each subgroup (negative, low/medium, and high expression).

**Figure 1:**
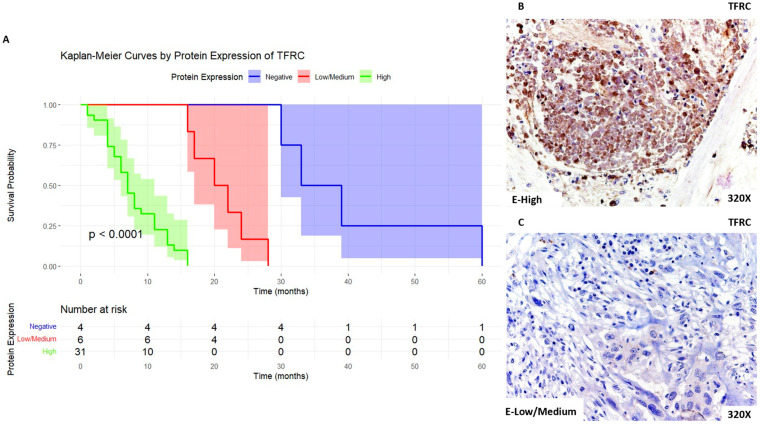
(**A**) Kaplan–Meier curves for survival time (months) according to tumor expression of TFRC. Blue curve: negative tissue expression; red curve: low/medium expression; green curve: high expression. (**B**,**C**) Histopathological images for high (intense brown staining) versus low/medium expression of TFRC.

We can observe that patients with high expression of TFRC present a median survival time of 7 [4.5–11] months, whereas those with low/medium expression exhibit a median survival time of 21 [17.8–23.5] months. Patients with negative expression of PDAC presented a median survival time of 36 [32.2–44.2] months. Figure 1A shows the Kaplan–Meier curve according to TFRC expression, whereas Figure 1B,C compare through IHC high expression levels of TFRC when compared to low/medium expression levels.

**Figure 2:**
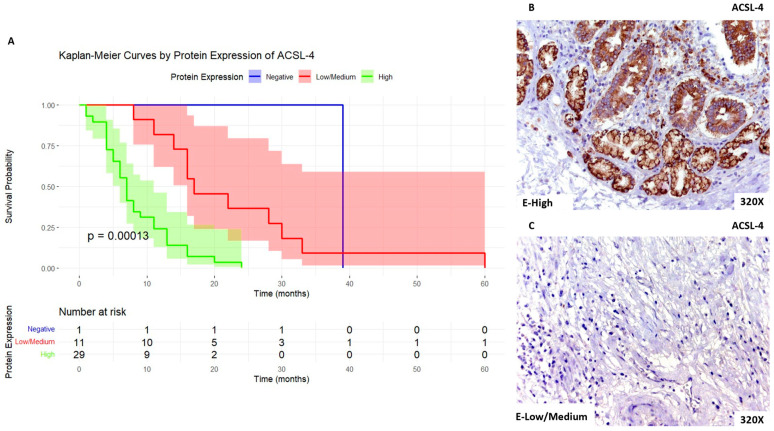
(**A**). Kaplan–Meier curves for survival time (months) according to tumor expression of ACSL-4. Blue curve: negative tissue expression; red curve: low/medium expression; green curve: high expression. (**B**,**C**). Histopathological images for high (intense brown staining) versus low/medium expression of ACSL-4.

We then report that patients with high expression of ACSL-4 present a median survival time of 7 [4–11] months, whereas those with low/medium expression exhibit a median survival time of 17 [15–29] months. Patients with negative expression of PDAC presented a median survival time of 39 [39–39] months. Figure 2A shows the Kaplan–Meier curve according to ACSL-4 expression, whereas Figure 2B,C compare through IHC high expression levels of ACSL-4 when compared to low/medium expression levels.

**Figure 3:**
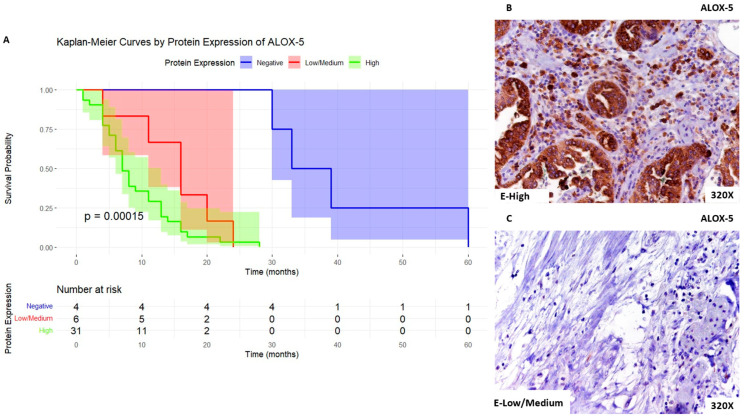
(**A**). Kaplan–Meier curves for survival time (months) according to tumor expression of ALOX-5. Blue curve: negative tissue expression; red curve: low/medium expression; green curve: high expression. (**B**,**C**). Histopathological images for high (intense brown staining) versus low/medium expression of ALOX-5.

Subsequently, our results show that patients with high expression of ALOX-5 present a median survival time of 7 [5–13] months, whereas those with low/medium expression exhibit a median survival time of 16 [12.2–19] months. Patients with negative expression of PDAC presented a median survival time of 36 [32.2–44.2] months. Figure 3A shows the Kaplan–Meier curve according to ALOX-5 expression, whereas Figure 3B,C compare through IHC high expression levels of ALOX-5 when compared to low/medium expression levels.

**Figure 4:**
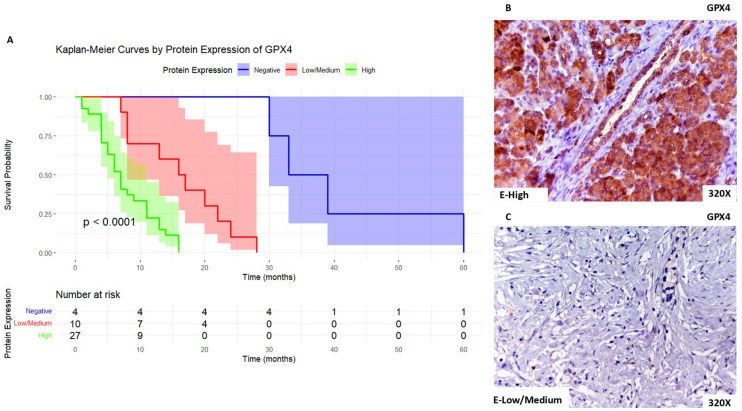
(**A**). Kaplan–Meier curves for survival time (months) according to tumor expression of GPX4. Blue curve: negative tissue expression; red curve: low/medium expression; green curve: high expression. (**B**,**C**). Histopathological images for high (intense brown staining) versus low/medium expression of GPX4.

We then report that patients with high expression of GPX4 exhibited a median survival time of 7 [4–11] months, whereas those with low/medium expression exhibit a median survival time of 16.5 [9.25–21.5] months. Patients with negative expression of PDAC presented a median survival time of 36 [32.2–42.2] months. Figure 4A shows the Kaplan–Meier curve according to GPX4 expression, whereas Figure 4B,C compare through IHC high expression levels of GPX4 when compared to low/medium expression levels.

On the other hand, we observed opposite patterns for circadian clock regulators and KLOTHO.

**Figure 5:**
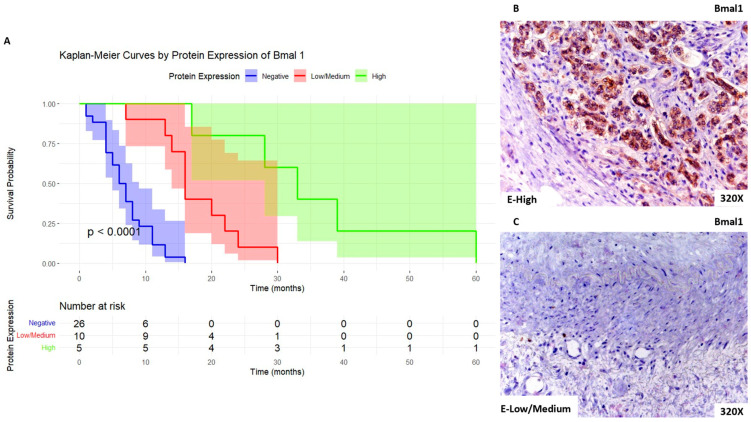
(**A**). Kaplan–Meier curves for survival time (months) according to tumor expression of Bmal 1. Blue curve: negative tissue expression; red curve: low/medium expression; green curve: high expression. (**B**,**C**). Histopathological images for high (intense brown staining) versus low/medium expression of Bmal1.

Regarding Bmal1, we can observe that patients with high expression levels of this marker show a median survival rate of 33 [28–39] months. In parallel, patients with low/medium expression present a median survival rate of 16 [14.5–21.5] months, whereas for negative expression of this marker, the median survival rate was 6.5 [4–8.75] months. Figure 5A shows the Kaplan–Meier curve according to Bmal1 expression, whereas Figure 5B,C compare through IHC high expression levels of Bmal1 when compared to low/medium expression levels.

**Figure 6:**
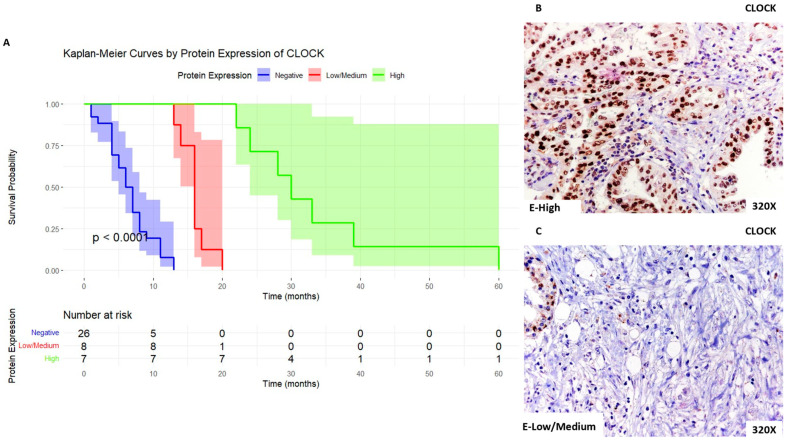
(**A**). Kaplan–Meier curves for survival time (months) according to tumor expression of CLOCK. Blue curve: negative tissue expression; red curve: low/medium expression; green curve: high expression. (**B**,**C**). Histopathological images for high (intense brown staining) versus low/medium expression of CLOCK.

Similarly, when we analyzed CLOCK expression, we report that patients with high expression levels of this marker show a median survival rate of 30 [26–36] months, whereas patients with low/medium expression present a median survival rate of 16 [15.5–16.2] months. Patients with negative expression of this marker presented a median survival rate of 6.5 [4–8] months. Figure 6A shows the Kaplan–Meier curve according to CLOCK expression, whereas Figure 6B,C compare through IHC high expression levels of CLOCK when compared to low/medium expression levels.

**Figure 7:**
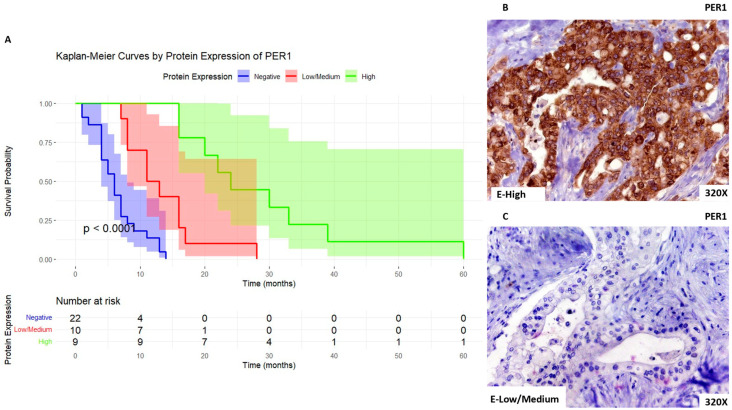
(**A**). Kaplan–Meier curves for survival time (months) according to tumor expression of PER1. Blue curve: negative tissue expression; red curve: low/medium expression; green curve: high expression. (**B**,**C**). Histopathological images for high (intense brown staining) versus low/medium expression of PER1.

When considering PER1, we can observe that patients with high expression levels of this marker show a median survival rate of 24 [20–33] months. In parallel, patients with low/medium expression present a median survival rate of 12 [8.75–16] months, whereas for negative expression of this marker the median survival rate was 6 [4–7.75] months. Figure 7A shows the Kaplan–Meier curve according to PER1 expression, whereas Figure 7B,C compare through IHC high expression levels of PER1 when compared to low/medium expression levels.

**Figure 8:**
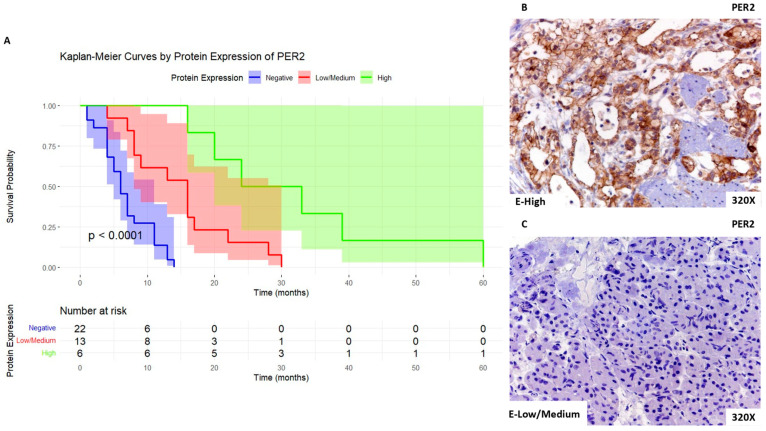
(**A**). Kaplan–Meier curves for survival time (months) according to tumor expression of PER2. Blue curve: negative tissue expression; red curve: low/medium expression; green curve: high expression. (**B**,**C**). Histopathological images for high (intense brown staining) versus low/medium expression of PER2.

Then, when we focused on PER2 expression, we report that patients with high expression levels of this marker show a median survival rate of 28.5 [21–37.5] months, whereas patients with low/medium expression present a median survival rate of 16 [8–17] months. Patients with negative expression of this marker presented a median survival rate of 6 [4–10.2] months. Figure 8A shows the Kaplan–Meier curve according to PER2 expression, whereas Figure 8B,C compare through IHC high expression levels of PER2 when compared to low/medium expression levels.

**Figure 9:**
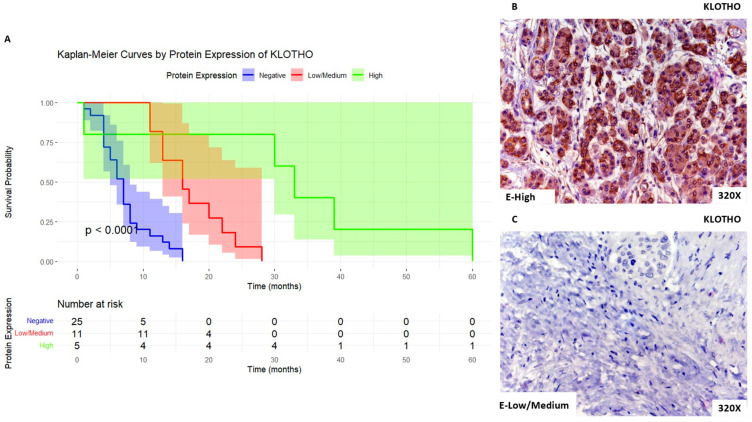
(**A**). Kaplan–Meier curves for survival time (months) according to tumor expression of KLOTHO. Blue curve: negative tissue expression; red curve: low/medium expression; green curve: high expression. (**B**,**C**). Histopathological images for high (intense brown staining) versus low/medium expression of KLOTHO.

Finally, we can observe that patients with high expression levels of KLOTHO show a median survival rate of 33 [30–39] months. In parallel, patients with low/medium expression present a median survival rate of 16 [13–21] months, whereas for negative expression of this marker, the median survival rate was 7 [4–8] months. Figure 9A shows the Kaplan–Meier curve according to KLOTHO expression, whereas Figure 9B,C compare through IHC high expression levels of KLOTHO when compared to low/medium expression levels.

#### 3.2.3. Evaluation of the Protein Markers at Predicting Survival Outcomes

A. **Log-Rank Test: Comparison of the survival distributions of different groups.**

In order to check if there are significant differences in survival between groups defined by different levels of protein expression, we developed a log-rank test for all the proteins included in the study, summarized in Supplementary Material S1 (SM1). As we can observe by our results, there is a statistically significant difference in survival between groups in our cohort defined by the expression levels of all ferroptosis and chronobiological markers, as well as KLOTHO (*** *p* < 0.001 for all cases).

B. **Cox Proportional Hazards Model: Estimation of the hazard ratio (HR) for each protein marker**

Once the statistical value of our model is demonstrated, we aim to define how the risk of the event (death) changes with each protein expression. With this aim, we have developed a hazard model for all the proteins studied, summarized in Table 5.

- We can observe that the ferroptosis markers (TFRC, ACSL-4, ALOX-5, GPX4) have HRs greater than 1, indicating an increased risk of death. The *p* values of the results obtained for these markers are statistically significative (*** *p* < 0.001). Of them, TFRC is the marker with the highest HR (35.905), suggesting the strongest association with increased risk. However, it should also be considered that the confidence interval (CI) of this marker is quite wide [4.859–265.3]. This means that the exact estimation of this effect may have some imprecision. The other ferroptosis markers (ACSL-4, ALOX-5, and GPX4) present HRs of 4.587, 3.826, and 7.738, respectively. However, since their CIs are lower, the magnitude of the mortality effect of these components is more certain than that obtained for TFRC.
- On the other hand, the chronobiological markers (CLOCK, Bmal1, PER1, PER2) and the antiaging marker (KLOTHO) have HRs significantly less than 1, indicating strong protective effects against death. The *p* values of the results obtained for these markers are also statistically significative (*** *p* < 0.001). Among these, CLOCK appears to have the most substantial protective effect (HR = 0.01832), followed by Bmal1 (HR = 0.1272). PER1, PER2, and KLOTHO presented an HR of 0.1922, 0.1872, and 0.1605, respectively.

### 3.3. Correlation Analysis

**Figure 10:**
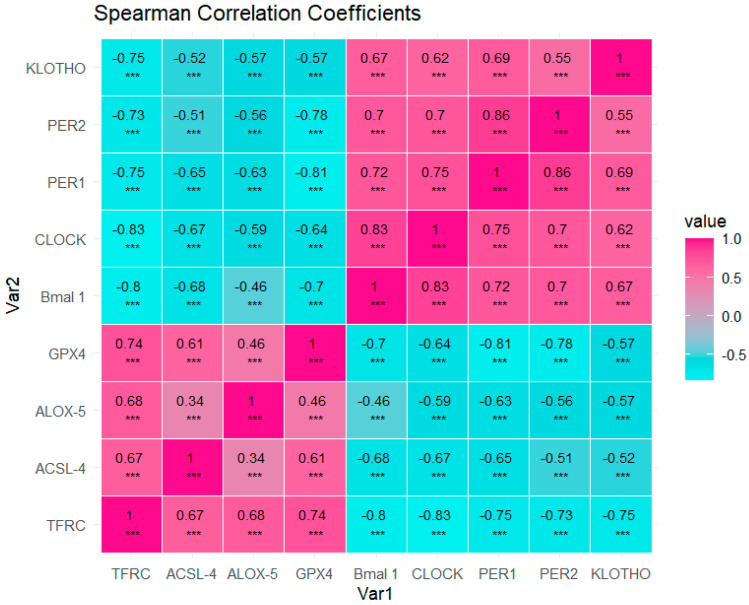
Pairwise Heatmap Matrix with Spearman correlation coefficients. *p* values associated to each Spearman correlation coefficient appear with asterisks and are adjusted by False Discovery Rate correction (FDR): adjusted alpha. We represent in cyan the variables with a protective role and in pink those that are risk markers. *** = *p* < 0.001.

By conducting a correlation analysis, we were able to examine the interrelationships among proteins using a correlation matrix (Figure 10). We applied the non-parametric analysis of Spearman and plotted the heatmap matrix with Spearman correlation coefficients and the asterisks of *p* values associated (*** *p* < 0.001). The *p* values are detailed in numbers in Supplementary Material S2 (SM2).

Our analysis revealed a notable positive correlation among ferroptosis markers, including TFRC, ACSL-4, ALOX-5, and GPX-4.

- We can observe that the strongest associations for ferroptosis markers were between TFRC with GPX-4, ALOX-5, and ACSL-4
- The weakest associations were observed for ALOX-5 with ACSL-4 and GPX4.

Similarly, circadian regulators such as Bmal1, CLOCK, PER1, and PER2 demonstrated a positive correlation amongst themselves, as well as with the protein KLOTHO.

- The strongest associations were observed between CLOCK with Bmal1 and PER1 as well as PER1 with PER2.
- The weakest associations were found between KLOTHO with PER2, CLOCK, and Bmal1.

Conversely, a negative correlation was observed between ferroptosis markers and circadian regulators, as well as with KLOTHO.

- The strongest inverse associations were observed between TFRC with CLOCK, Bmal1, PER1, KLOTHO, and PER2.
- The weakest associations were found between Bmal1 and ALOX-5, as well as ACSL-4 with PER-2 and KLOTHO

### 3.4. Evaluation of Categorical Variables with Multinomial Logistic Regression (MLR)

Before performing MLR Analysis, we applied Fisher’s Exact Test for the statistical treatment of categorical predictive variables (sociodemographic data) to evaluate their significance in the expression of the protein markers. Through Supplementary Material S3 (SM3), we justified the adjusted models chosen. Afterwards, to diminish the bias of this test and the low sample size (N = 41), we applied FDR correction of Benjamini–Hochberg to diminish random chance (Supplementary Material S4, SM4). Due to the repetition of “chronic pathologies” as the significant variable either alone as well as in combination with the others, before arriving at the following step of MLR models, we also crossed as a survival Kaplan–Meier analysis (Supplementary Material S5, SM5) this variable with the survival time to see if there is a significant relationship. Through log-rank test, we obtained a *p* value of 0.086, seeing that there is no statistical significance among these data (SM5).

Our MLR models (Table 6) show that the presence of chronic pathologies has an impact on the likelihood to change the expression levels of several protein markers.

Certainly, there are only 4 patients out of 41 (N) who have a chronic pathology; therefore, the significance may be due to chance, and we cannot assure if this condition is truly affecting the prognosis of the expression of the protein markers. Even though we found statistical significance (*p* < 0.05 *) through Fisher’s Exact Test and then its correction through FDR, between the predictive variable “chronic pathology” (alone and in combination with the rest of the sociodemographic variables) and a great part of the proteins, we did not find too much significance in the MLR models. We found significance (*p* < 0.05 *) for the presence of chronic comorbidity in the expression of TFRC, GPX4, Bmal 1, CLOCK, and KLOTHO, in all cases comparing the baseline with the high expression of each of these proteins:

- TFRC odds ratio < 1 (0.033) indicates that individuals are significantly less likely to transition to higher expression levels of TFRC. The odds of transitioning to higher levels are reduced by 96.7% (1 − 0.033). This result is statistically significant, indicating a strong reduction in the likelihood of increasing TFRC expression levels.
- GPX4 odds ratio < 1 (0.03845) indicates that individuals are significantly less likely to transition to higher expression levels of GPX4. The odds of transitioning to higher levels are reduced by 96.155% (1 − 0.03845). This result is statistically significant, indicating a strong reduction in the likelihood of increasing GPX4 expression levels.
- Bmal 1 odds ratio > 1 (16.667) indicates that individuals are significantly more likely to transition to higher expression levels of Bmal 1. The odds of transitioning to higher levels are increased by 1566.7% (16.667 − 1). This result is statistically significant, indicating a strong increase in the likelihood of increasing Bmal 1 expression levels.
- CLOCK odds ratio > 1 (18.75) indicates that individuals are significantly more likely to transition to higher expression levels of CLOCK. The odds of transitioning to higher levels are increased by 1775% (18.75 − 1). This result is statistically significant, indicating a strong increase in the likelihood of increasing CLOCK expression levels.
- KLOTHO odds ratio > 1 (15.99916) indicates that individuals are significantly more likely to transition to higher expression levels of KLOTHO. The odds of transitioning to higher levels are increased by 1499.916% (15.99916 − 1). This result is statistically significant, indicating a strong increase in the likelihood of increasing KLOTHO expression levels.

In summary, these results suggest that, independently from the rest of the sociodemographic conditions, a person with a chronic pathology has less probability of expressing high levels of ferroptosis markers and more probability of expressing high levels of chronobiological markers.

We can hypothesize for future studies that having a chronic disease can affect the level of expression of each protein marker and therefore the prognosis of patients.

### 3.5. Evaluation of Numerical Clinical Variables with Kruskal–Wallis Test and Ordinal Logistic Regression Models (OLRs)

Through Kruskal–Wallis test, we found the relationship between CLOCK levels of expression and age to be statistically significant (Table 7). We also found statistical significances in the levels of expression of GPX4, Bmal 1, PER1, and PER2 with the measure of Ca 19-9, particularly high levels of this carcinogenic antigen.

We performed further analysis with the Ordinal Logistic Regression (OLR) models to check if those clinical parameters could predict protein expression levels (Table 8). Age does not appear to be a statistically significant predictor of KLOTHO protein expression levels in our sample. However, the elevated levels of carcinogenic marker Ca 19-9 did show statistical significance for the transitioning from a level of expression to the following advanced level for several protein markers.

- In the case of GPX4, it resulted as significant (*p* < 0.01 **) the ODDs < 1 (0.22) for transitioning from negative to low/medium expression, meaning that it is unlikely for individuals to transition to medium expression levels. Specifically, the odds of this transition are reduced by 78% (1 − 0.22). The result is statistically significant, suggesting that there is a strong, significant reduction in the likelihood of moving to low/medium expression levels from negative expression levels.
- For Bmal 1, it resulted as significant (*p* < 0.05 *) the ODDs > 1 (3.17) for transitioning from low/medium to high expression, meaning that it is likely for individuals to transition to higher expression levels. The odds of this transition are increased by 217% (3.17 − 1). This result is statistically significant, suggesting a significant increase in the likelihood of moving to high expression levels from low/medium expression levels.
- In the case of PER1, it resulted as significant (*p* < 0.05 *) the ODDs > 1 (2.29) for transitioning from low/medium to high expression, meaning that there is an increase in the likelihood for individuals to transition to higher expression levels. The odds of this transition are increased by 129% (2.29 − 1). This result suggests a significant increase in the likelihood of moving to high expression levels from low/medium expression levels.
- Finally, for PER2, it resulted as significant (*p* < 0.05 *) the ODDs < 1 (0.35) for transitioning from negative to low/medium expression, meaning that it is unlikely for individuals to transition to medium expression levels. The odds of this transition are reduced by 65% (1 − 0.35). This result suggests a significant reduction in the likelihood of moving to low/medium expression levels from negative expression levels. Also, it resulted as significant (*p* < 0.05 *) the ODDs > 1 (2.67) for transitioning from low/medium to high expression, meaning that it is likely for individuals to transition to higher expression levels. The odds of this transition are increased by 167% (2.67 − 1). This result suggests a significant increase in the likelihood of moving to high expression levels from low/medium expression levels.

See in detail in Table 8. 

## 4. Discussion

In the present work, we have observed a direct association between increased protein expression of ferroptosis markers (TFRC, ACSL-4, ALOX-5, and GPX4) along with an inverse correlation between circadian clock regulators (CLOCK, Bmal1, PER1, and PER2) and KLOTHO with PDAC mortality. Our statistical analysis shows that TFRC and CLOCK were the proteins more clearly related to PDAC mortality; however, whereas TFRC markers show a direct association (risk factor), CLOCK expression shows an inverse association (protective factor). Additionally, our correlation analysis shows interesting biological connections between these molecules that should be further explored. Finally, our multinomial logistic regression model shows that among the different clinical variables included in our study, only the presence of chronic pathologies seems to be associated with the expression of the different molecules included in our study. Additionally, we also report differential expression patterns according to its combination with additional clinical variables explored, suggesting that the presence of baseline chronic pathologies may partly influence PDAC biology and tumor development. To provide a clearer exposition and discussion of our results we will divide this section into different subparts.

### 4.1. Augmented Expression of Ferroptosis Markers Is Associated with Lower Survival Rates in Patients with Pancreatic Ductal Adenocarcinoma

Firstly, we have observed that the enhanced expression of ferroptosis markers seems to have a significant negative impact on PDAC survival. Liu et al. [20] defined that ferroptosis could be intricately linked to the decreased survival and carcinogenesis of PDAC by increasing the release of damage-associated molecular pattern molecules (DAMPs), triggering an inflammatory response that promotes tumor growth and development. Apart from its relationship with inflammation, iron accumulation is an important source of reactive oxygen species (ROS) and reactive nitrogen species (RNS) through the Fenton reaction leading to the subsequent oxidative stress, whereas, in turn, oxidative stress and lipid peroxidation are important inducers of ferroptosis [39]. Ferroptosis is also tightly related to autophagy, and this interaction could be potentially involved in PDAC development [40]. Importantly, prior works have connected increased expression of oxidative stress, autophagy, and inflammation markers with poor survival in PDAC patients [7,38,41]. The association of ferroptosis with those mechanisms could partly explain its relationship with poor PDAC prognosis. However, it is important to consider that ferroptosis seems to play a dual role in PDAC development and progression according to the characteristics of the tumor microenvironment and mutations [19].

In our retrospective cohort, TFRC, ALOX-5, ACSL-4, and GPX-4 have shown a direct correlation with PDAC mortality, with TFRC being the component more clearly associated with this. TFRC is involved in the regulation of cellular uptake and entry of iron [42]. The relevance of this marker in PDAC has been supported in past works. Ryschich et al. [43] reported that TFRC expression represented a marker of malignization in the pancreas that could be potentially used as a biomarker or therapeutic target. In a similar line, Yang et al. [23] also observed that increased expression of TFRC was associated with a poor prognosis in three patients with PDAC, with an HR of 1.681. With their bioinformatics analysis, they showed that TFRC might play a role in the occurrence and development of PDAC mainly through signaling pathways (including cell adhesion molecule binding, condensed chromosomes, chromosome segregation, and cell cycle checkpoints) and through its association with immune phenotypes and immune cell infiltration. In our study including 41 patients with PDAC, we observe that TFRC is more clearly associated with mortality with an HR of 35.905. Based on our results and the available studies, we suggest that TFRC is an important biomarker of PDAC potentially involved in the carcinogenesis of this entity, evidencing the need for future works evaluating other potential applications of this component (for instance, as a predictive marker or therapeutic target).

On the other hand, ALOX-5 and ACSL-4 are two critical enzymes involved in ferroptosis augmented in our study. ALOX-5 is an iron-containing, nonheme dioxygenase that catalyzes the peroxidation of polyunsaturated fatty acids (PUFAs) such as arachidonic acid, being implicated in the biosynthesis of leukotrienes, the modulation of the inflammatory responses, and various types of cell death—such as apoptosis, pyroptosis, and the proper ferroptosis [44,45]. On the other hand, ACSL4 is an enzyme involved in the enrichment of cellular membranes with long omega-6 PUFAs, thereby increasing the sensitivity of the cell to trigger ferroptosis [46]. The role of both enzymes in PDAC has been demonstrated in past research. Various studies have found that ALOX-5 could play a central role in therapy resistance in PDAC, representing an interesting biomarker and therapeutic target to consider [47,48,49]. The relevance of ACSL-4 in PDAC is starting to be elucidated, although most studies and related conclusions have been drawn in vitro [50,51,52]. Bai et al. [53] identified 39 deferentially expressed genes in 179 pancreatic cancer samples, ACSL4 being one of the 36 upregulated genes reported. However, their bioinformatic analysis did not show any prognostic association of this molecule. In our study, both ACSL-4 and ALOX-5 show a direct association with PDAC mortality, with an HR of 4.587 and 3.826, respectively. Future works deepening on the mechanistic action of both ferroptosis-related molecules and possible translational applications derived should be performed in future works.

Finally, another key regulator of ferroptosis, GPX4, was also upregulated in our cohort, having a direct effect on mortality with an HR of 7.738. GPX4 is an antioxidant acting together with reduced glutathione (GSH) and α-tocopherol as antiperoxidative mechanism, limiting processes of lipid peroxidation and ferroptosis [54]. Specifically, GPX4 is selenoenzyme-implicated in the reduction of phospholipid hydroperoxides (PLOOHs) [55]. Past works have demonstrated that increased expression of this component may collaborate with PDAC therapy resistance by limiting the process of ferroptosis [56,57]. Dai et al. [58] reported that PDAC exhibits increased GPX4 expression when compared with normal adjacent tissue, also reporting that this marker represents a valuable prognostic biomarker. However, contrary to our results, they show that patients with high expression of GPX4 showed better prognosis than those with lower expression. Future works should be performed to clarify the prognostic value of GPX-4, considering possible clinical and biological variables that may explain these differences.

### 4.2. Reduced Expression of Circadian Regulators Is Related to Lower Survival Rates in Patients with Pancreatic Ductal Adenocarcinoma

We then observed that circadian clock regulators have shown an inverse association with PDAC mortality in our cohort sample. In our study, we observed that CLOCK was the protein more clearly related to increased mortality in PDAC subjects (HR = 0.01832), followed by Bmal1 (HR = 0.1272) and PER1/2 (HR = 0.1922 and 0.1872, respectively). The circadian rhythm is a 24-h internal clock in our brain that regulates alertness and sleepiness by responding to environmental light changes, shaping our physiology and behavior according to the Earth’s rotation. This biological system has evolved to help humans adapt to environmental changes and anticipate variations in radiation, temperature, and food availability, optimizing energy expenditure and internal physiology [59]. There are various molecules implicated in the regulation of circadian rhythms. In a very simple manner, CLOCK and BMAL1 form a heterodimeric complex that activates the transcription of clock genes by binding to E-box sequences in their promoters, regulating the expression of different genes involved in metabolic, biosynthetic, signal transduction, and cell cycle pathways [60]. In turn, CLOCK and Bmal1 also regulates the expression of key genes implicated in their proper regulation such as PER1, PER2, and PER3 and cryptochromes CRY1 and CRY2. PER and CRY proteins form a complex in the cytoplasm that translocates into the nucleus inhibiting the transcriptional activity of the BMAL1: CLOCK complex [60]. Tissue expression of CLOCK and Bmal1 is regulated by a central clock located in the suprachiasmatic nucleus of the hypothalamus (SCN), which is responsible for receiving and integrating environmental signals (light exposure) and controlling extra-SCN clocks in other brain regions via rhythmic release of neurotransmitters and neuropeptides and of peripheral tissues via systemic hormonal secretion and neural innervation [61]. Therefore, CLOCK, Bmal1, PER1, and PER2 are part of an autoregulatory loop involved in the regulation of circadian rhythms, being controlled by a central clock formed by the NSQ and other brain regions in response to environmental signals.

Loss of circadian regulation has been associated with a plethora of systemic maladies, including cancer [62,63,64]. An aberrant circadian clock functioning seems to play an important role in tumorigenesis, promoting tumor growth, metastasis, immune evasion, and other processes by regulating various biological processes apoptosis and proliferation. From a molecular perspective, these effects are related to significant changes in many signaling pathways and critical components involved in cancer regulation such as the AMPK/mTOR pathway, Wnt/β-Catenin pathway, NF-κ B pathway, HIF-1α, P53, and PD-1 [65]. In vitro and in vivo models demonstrated that circadian clock disruption is linked to the development and progression of PDAC, as well as therapy resistance [66].

In a recent work, Schwartz et al. [30] demonstrated that Bmal1, CLOCK, PER1, and PER2, together with other circadian clock regulators, are significantly altered in PDAC cell lines. In agreement with our observations, they reported that decreased Bmal1 expression was associated with a poor prognosis in PDAC patients, also suggesting that altered expression of CLOCK, PER1, and PER2 may have a significant impact on this variable to be explored yet. In a similar line, Relles et al. [67] also found that gene expression levels of several circadian proteins like Per1, Per2, CLOCK, and BMAL1 were decreased in PDAC tumors when compared to normal adjacent pancreatic tissue, the downregulation of these molecules being associated with a poorer prognosis.

The role of CLOCK in PDAC is not currently understood yet; however, this protein together with Bmal1 controls a broad spectrum of carcinogenic processes and pancreatic function, as detailed by García-Costela et al. [66]. According to past works, Bmal1 can act as a potent anti-oncogene in PDAC by activating the downstream p53-dependent tumor suppressor pathway [68], this component being considered an independent prognostic factor for tumor progression and poor survival outcome for patients with PDAC [69]. Similarly, PER2 overexpression in human cell lines of pancreatic cancer showed reduced cellular proliferation and induced apoptotic cell death and cell cycle arrest at the G(2)-M phase [70]. Interestingly, this effect could enhance the sensitivity to cisplatin depending on Bcl-X(L) expression level. Because of this, Tavano et al. [71] evidenced after adjusting for several variables that patients with higher PER2 and lower sirtuin 1 (SIRT1) expression levels showed lower mortality, remarking the relevance of PER2 as a prognostic factor. Past works also report that PER-1 downregulation could be an important carcinogenic mechanism of PDAC [72,73]; however, one study [74] reported that this component appeared to be increased in pancreatic cancer tissue, possibly acting as an anti-apoptotic factor in pancreatic cancer cells. Future works should be directed in order to understand the carcinogenic and potential translational implications of these molecules in PDAC.

### 4.3. Decreased Expression of Klotho Seems to Be a Marker of Poor Prognosis in Patients with Pancreatic Ductal Adenocarcinoma

Lastly, our results show that protein expression of KLOTHO was significantly related to PDAC prognosis in our cohort sample. In more detail, higher expression levels of this marker were associated with a better prognosis and reduced mortality risk (HR = 0.1605). KLOTHO was initially seen as an “aging-suppressor” gene in mice, which accelerates aging when disrupted and extends lifespan when overexpressed [75]. Multiple KLOTHO protein forms have been characterized: α-Klotho, β-Klotho, and γ-Klotho, with different actions and expression across tissues [76]. Additionally, there is a full-length transmembrane KLOTHO (mKL), two truncated soluble Klotho forms, and a secreted Klotho (sKL) form, [77]. The Klotho proteins are vital for enabling high-affinity binding of endocrine fibroblast growth factor 19 (FGF19), FGF21, and FGF23 to their FGF receptors, collectively forming an endocrine system that regulates various metabolic processes in mammals [78].

Downregulation of KLOTHO by different epigenetic mechanisms has been reported in several types of cancer, resulting in aberrations in FGF signaling, as well as disturbed insulin-like growth factor 1 receptor (IGF-1R) and the Wnt/β-catenin signaling pathway [33]. Thus, the literature considers KLOTHO as a critical tumor suppressor, influencing in cell proliferation, survival, autophagy, and resistance to anti-cancer therapies [79]. In agreement with this fact, Rubinstein et al. [80] also suggest that KLOTHO is a tumor suppressor in PDAC, suggesting that this could represent a potential prognostic marker in patients with pancreatic cancer. Jiang et al. [81] also found that decreased expression of Klotho was associated with reduced survival and high clinical and pathological stages in PDAC patients, claiming an existing correlation between miR-504 levels with Klotho mRNA and promoter methylation. Thus, our study also supports the prognostic value of KLOTHO in our cohort sample. This could open potential lines of research regarding the use of this protein in clinical routine. In this sense, the therapeutic use of Klotho, Klotho-derived peptides, or its subdomain KL1 formed by cleavage or alternative splicing in PDAC has also been supported in vitro and in vivo [82,83]. Future studies should be directed to deepen on the multiple translational applications of Klotho in PDAC.

### 4.4. Protein Expression of Ferroptosis Markers Show an Inverse Correlation with Circadian Regulators and Klotho

In our investigation, we employed a comprehensive approach to assess the potential correlations between protein expressions of ferroptosis markers (TFRC, ALOX-5, ACSL-4, and GPX-4) and circadian regulators (CLOCK, PER1, PER2, and BMAL1), as well as KLOTHO, within the context of PDAC. Utilizing a correlogram analysis, we successfully demonstrated significant associations among these molecular entities, indicating potential interplay or regulatory relationships within the tumoral environment. Firstly, we observed that ferroptosis markers show a direct correlation between them, as occurs with circadian clock regulators which also show a positive correlation with the expression of KLOTHO. Conversely, both KLOTHO and circadian regulators show inverse correlation patterns with ferroptosis markers.

A growing body of evidence is starting to support the existing opposite relationship between ferroptosis and circadian clock regulators. However, to date, it is difficult to establish a precise molecular connection between ferroptosis and circadian regulators in PDAC, due to the low number of available studies. Some interesting studies in the past have suggested possible pathways that should be explored in the future. We report that the strongest correlations between ferroptosis and chronobiological markers can be observed between TFRC-CLOCK and TFRC-Bmal1. Previous works have evidenced that TFRC expression seems to follow a 24-h rhythm in mRNA and protein levels in colorectal cancer cells, particularly through the oncogene c-myc [84]. C-myc is a clock-controlled gene commonly overexpressed in PDAC tumors, acting as a marker of poor prognosis [85]. C-myc gene is directly suppressed by CLOCK:BMAL1 since its promoter possess E-boxes and is stabilized by PER1 [66], although it can also been regulated by other mechanisms. It is possible that the lower levels of circadian clock regulators contribute to a dysregulation and increased expression of c-myc, leading to enhanced expression of TFRC. Further studies are however warranted to prove this hypothesis.

On the other hand, various works [86,87] have found that the selective autophagic degradation of the circadian clock regulators (mainly BMAL1) named as clockophagy can promote ferroptotic cancer cell death in vitro and in vivo. Past works have also demonstrated that Bmal1 deletion was associated with cellular iron overload and ferroptosis [88]. In a similar way, Liu et al. [89] have also found that Bmal1 is able to protect against experimental acute pancreatitis through blocking the ferroptosis-mediated release of HMGB1, a mediator of sterile inflammation, while promoting the expression of multiple antioxidant or membrane repair systems, thereby suppressing ferroptosis-mediated damage in pancreatic tissue. The co-occurrence of ferroptosis with the exacerbation of inflammatory signaling pathways like JAK-STAT, NF-κB, NLRP3 inflammasome, cGAS-STING, and MAPK signaling pathways has been defined in past works [90], whereas an inverse association between inflammatory pathways and circadian genes has also been reported [91]. We previously explored that overexpression of the NLRP3 inflammasome is directly correlated with poor prognosis in PDAC [9]. As a growing body of evidence suggests that this component can impair the circadian clock [92], it would be interesting to determine possible carcinogenic connections between both elements. Therefore, exploring the link between ferroptosis and circadian dysregulation through inflammation may be an important point of study for future works.

In addition, the studies have also explored multiple associations between oxidative stress, ferroptosis, and chronobiology. As previously mentioned, oxidative stress and lipid peroxidation are tightly linked to ferroptosis, but compelling evidence has also shown that oxidative damage, such as protein oxidation or lipid peroxidation, exhibits circadian patterns, with some circadian proteins like PER1/2 playing a major role in this regulation in animal models [93]. Similarly, tissue expression of chronobiological markers like PER1 was found to be inversely correlated with ferroptosis markers like GPX4 in human nasopharyngeal carcinoma (NPC) cell line (CNE2) and NPC tissues [94]. The aforementioned SIRT-1 is also a potential molecule that should be explored in this sense. SIRT-1 is a molecule implicated in oxidative stress through the initiation of several downstream effectors, including p53 and FOXO transcription factors, which is also able to regulate circadian rhythms by deacetylating clock proteins BMAL1 and PER2 [93]. Additionally, SIRT-1 is a molecule regulated by circadian modulators like melatonin and NAD+ availability, linking the redox state to circadian regulation. The role of SIRT-1 in PDAC carcinogenesis has been demonstrated in past works [95], showing an inverse association with PDAC survival [71]. Likewise, there is a direct link between the redox state of NAD+/NADH and NADP+/NADPH with circadian rhythms, affecting the binding of circadian transcription factors [96]. NADPH oxidases (NOX) are crucial enzymes that overproduce O2− inside the cell and influence the redox state. NOX-1 and NOX-2 have been shown to be overexpressed in PDAC tumors, leading to enhanced oxidative stress and showing a direct association with mortality [7]. Overall, oxidative stress may be a pivotal mechanism linking ferroptosis and circadian disruption in PDAC tumors, although further efforts unveiling these associations are needed. Likewise, future studies evaluating the influence of circadian regulators like Bmal1 or CLOCK in PDAC are still warranted to establish possible molecular networks in these tumors.

**Figure 11:**
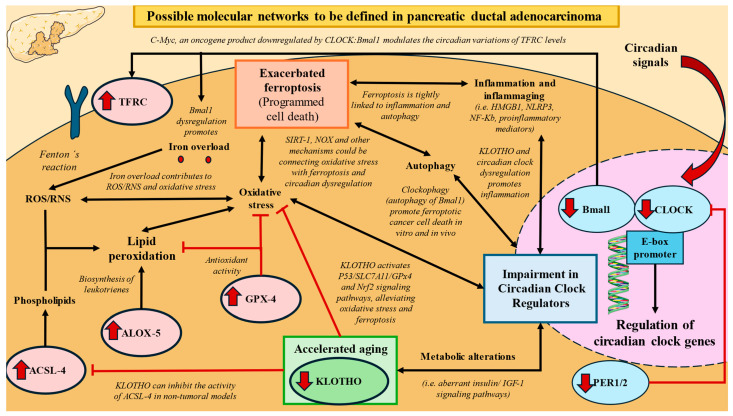
A graphical summary of our results and proposed biological networks that may be acting. Red arrows represent inhibitory effects, black arrows defines established bidirectional or unidirectional associations.

On the other hand, the literature also recognized an inverse association between the protein KLOTHO and ferroptosis. Indeed, KLOTHO expression is able to suppress ferroptosis in various tissues, as demonstrated in in vitro and in vivo models [97,98,99]. Despite not explored in cancer, molecular links between KLOTHO and inhibition of ferroptosis have been associated in previous works with the regulation of the nuclear erythroid 2-related factor 2 (Nrf2) and P53/SLC7A11/GPx4 signaling pathways, being also able to inhibit other components such as ACSL4 [98,99,100]. The relationship between KLOTHO and circadian clock, however, is less established in the available literature. However, as both circadian regulators and KLOTHO are critical molecules implicated in various health, antiaging, and anticarcinogenic processes [33,78,101], a possible direct or indirect association between them could be occurring in PDAC. A really interesting link to be explored between KLOTHO and circadian clock includes their association and effects on energy metabolism. For providing an example, not only KLOTHO but also circadian clock components interact with insulin/insulin growth factor-1 signaling pathways [102,103,104], whose relevance in PDAC carcinogenesis and prognosis has been previously demonstrated [105,106]. Likewise, KLOTHO is able to modulate oxidative stress through modulating signaling pathways involving antioxidants and Nrf2 [107], also mitigating various inflammatory cytokines and products [108]. Overall, this integrated analysis sheds light on the intricate molecular landscape underlying PDAC progression, although broader efforts are needed for understanding the precise mechanisms potentially found in this association. In Figure 11, we propose potential mechanisms to be explored which could explain the association between exacerbated ferroptosis, impaired circadian clock regulators, and accelerated aging presented by downregulation of KLOTHO.

### 4.5. The Presence of Chronic Pathologies Was the Unique Clinical Variable Correlated with the Expression of the Analyzed Proteins

On the other hand, when we considered the individual protein expressions of these markers in correlation with various clinical parameters—including smoking, drinking, sex, prior malignancies, obesity/diabesity, and chronic pathologies—we only observed statistically significant associations with the presence of chronic pathologies. Certainly, there are only 4 patients out of 41 who have a chronic pathology; therefore, the significance may be due to chance, and we cannot assure if this condition is truly affecting the prognosis of the expression of the protein markers. Even though we found statistical significance (*p* < 0.05 *) through Fisher’s Exact Test and then its correction through FDR, between the predictive variable “chronic pathology” (alone and in combination with the rest of the sociodemographic variables) and a great part of the proteins, we did not find too much significance in the MLR models. We found significance (*p* < 0.05 *) for the presence of chronic comorbidity in the expression of TFRC, GPX4, Bmal 1, CLOCK, and KLOTHO, in all cases comparing the baseline with the high expression of each of these proteins. TFRC odds ratio (0.033 < 1). GPX4 (0.03845 < 1), Bmal 1 (16.667 > 1), CLOCK (18.75 > 1), KLOTHO (15.99916 > 1). While clinical parameters undoubtedly play a crucial role in patient outcomes and treatment responses, our study suggests that apart from chronic pathologies, the regulation of ferroptosis markers, circadian regulators, and KLOTHO in PDAC may be governed by other factors not captured in our clinical parameter analysis.

Overall, our findings contribute to a deeper understanding of the molecular intricacies underlying PDAC and underscore the importance of considering multifactorial interactions in elucidating disease mechanisms and identifying potential therapeutic targets. Further studies exploring additional molecular pathways and their interactions with clinical parameters are warranted to enhance our understanding and improve patient outcomes in PDAC management.

## 5. Conclusions

In the present work, the prognostic value of ferroptosis markers (TFRC, ALOX-5, ACSL-4, and GPX-4), circadian clock regulators (CLOCK, Bmal1, PER1, PER2), and KLOTHO is demonstrated in a retrospective cohort of 41 patients with PDAC. In more detail, the explored ferroptosis markers show a direct association with PDAC mortality, whereas oppositely, circadian regulators and KLOTHO show an inverse association. TFRC was the risk marker more clearly associated with mortality (HR = 35.905) and CLOCK the protective marker more clearly related to mortality (HR = 0.01832). Our correlation analyses show that ferroptosis markers show a direct correlation among themselves, similar to circadian clock regulators, which also positively correlate with KLOTHO expression; conversely, both KLOTHO and circadian regulators exhibit inverse correlation patterns with ferroptosis markers. Among the different clinical variables included in the present study, only the presence of chronic pathologies was associated with the expression patterns of various proteins included in our study. These observations underscore the complex nature of PDAC pathogenesis and highlight the need for further investigation into the specific molecular mechanisms driving disease progression.

## Figures and Tables

**Table 1:** Primary and secondary antibodies used and their dilutions.

Antigen	Species	Dilution	Provider	Protocol Specifications
TFRC	Rabbit Monoclonal	1:500	Abcam (Cambridge, UK) ab185550	EDTA pH = 9 before incubation with blocking solution
ACSL-4	Rabbit Monoclonal	1:100	Abcam (Cambridge, UK) ab155282	100% Triton 0.1% in PBS, 10 min, before incubation with blocking solution
ALOX-5	Rabbit Monoclonal	1:250	Abcam (Cambridge, UK) ab169755	100% Triton 0.1% in PBS, 10 min, before incubation with blocking solution
GPX4	Rabbit Monoclonal	1:100	Abcam (Cambridge, UK) ab125066	10 mM of Sodium citrate pH = 6 before incubation with blocking solution
BMAL1	Rabbit monoclonal	1:1000	Abcam (Cambridge, UK) ab230822	-
CLOCK	Rabbit policlonal	1:100	Abcam (Cambridge, UK) ab3517	-
PER1	Rabbit polyclonal	1:20	Abcam (Cambridge, UK) ab254751	-
PER2	Rabbit polyclonal	1:100	Abcam (Cambridge, UK) ab200388	-
KLOTHO	Rabbit monoclonal	1:100	Abcam (Cambridge, UK) ab181373	-
IgG (Rabbit)	Mouse	1:1000	Sigma-Aldrich (Burlington, USA) RG-96/B5283	-

**Table 2:** Clinical and sociodemographic characteristics of the evaluated cohort of pancreatic cancer.

Variable	n (%)
**Numeric (median (IQR)**	
Age	72 (45–88)
**Categoric n (%)**	
Sex (women)	14 (34.15)
Smoking	18 (43.90)
Drinking	11 (26.83)
Obesity	2 (4.88)
Type 2 Diabetes + Obesity (Diabesity)	15 (36.59)
Chronic pathologies	4 (9.76)
Prior Malignances	11 (26.86)
Stage IV (in the moment of diagnosis)	13 (31.71)

IQR: interquartile range. n: number. N = 41 (total cohort size).

**Table 3:** Plasma levels of the main carcinogenic markers collected routinely (values are expressed as a median and interquartile range).

Marker (Reference Values)	Median [IQR]
Ca 19-9 U/mL (0–37)	102.10 [44.91–805.00]
CEA ng/mL (0–5)	5.43 [2.71–11.31]
AFP ng/mL (0–13.4)	2.32 [1.46–4.39]

IQR: interquartile range. Ca 19-9 = Carbohydrate antigen. CEA = Carcinoma embryonic antigen. AFP = Alpha-Fetoprotein. N = 41 (total cohort size).

**Table 4:** Median time of survival (months) and interquartile range (IQR) for each protein marker.

Marker (n)	Median Time	IQR (Q1–Q3)
**Ferroptosis markers**		
**TFRC**		
Negative (4)	36	32.2–44.2
Low–Medium (6)	21	17.8–23.5
High (31)	7	4.5–11
**ACSL-4**		
Negative (1)	39	39–39
Low–Medium (11)	17	15–29
High (29)	7	4–11
**ALOX-5**		
Negative (4)	36	32.2–44.2
Low–Medium (6)	16	12.2–19
High (31)	7	5–13
**GPX4**		
Negative (4)	36	32.2–44.2
Low–Medium (10)	16.5	9.25–21.5
High (27)	7	4–11
**Chronobiological markers**		
**Bmal 1**		
Negative (26)	6.5	4–8.75
Low–Medium (10)	16	14.5–21.5
High (5)	33	28–39
**CLOCK**		
Negative (26)	6.5	4–8
Low–Medium (8)	16	15.5–16.2
High (7)	30	26–36
**PER1**		
Negative (22)	6	4–7.75
Low–Medium (10)	12	8.75–16
High (9)	24	20–33
**PER2**		
Negative (22)	6	4–10.2
Low–Medium (13)	16	8–17
High (6)	28.5	21–37.5
**Antiaging markers**		
**KLOTHO**		
Negative (25)	7	4–8
Low–Medium (11)	16	13–21
High (5)	33	30–39

IQR: interquartile range; N = 41 (total cohort of patients with pancreatic cancer).

**Table 5:** Hazards model for all the proteins.

Protein Marker	Hazard Ratio (HR)	95% Confidence Interval (CI)	*p* Value	Interpretation
**Ferroptosis markers**				
TFRC	35.905	[4.859–265.3]	0.00045 ***	High HR indicates a very strong association with increased risk of death. However, the wide CI suggests some uncertainty in the exact magnitude of the effect.
ACSL-4	4.587	[2.087–10.08]	0.00015 ***	Significantly increases risk of death.
ALOX-5	3.826	[1.846–7.927]	0.000307 ***	Significantly increases risk of death.
GPX4	7.738	[3.061–19.56]	1.53 × 10^−5^ ***	Significantly increases risk of death.
**Chronobiological markers**				
Bmal 1	0.1272	[0.05679–0.285]	5.42 × 10^−7^ ***	Strong protective effect against death.
CLOCK	0.01832	[0.00245–0.137]	9.74 × 10^−5^ ***	Very strong protective effect against death.
PER1	0.1922	[0.1013–0.3646]	4.44 × 10^−7^ ***	Strong protective effect against death.
PER2	0.1872	[0.09209–0.3804]	3.65 × 10^−6^ ***	Strong protective effect against death.
**Antiaging markers**				
KLOTHO	0.1605	[0.07389–0.3485]	3.77 × 10^−6^ ***	Strong protective effect against death.

***Interpretation example***: These values indicate that the hazard (or risk) of the event (e.g., death) for patients with higher TFRC expression is about 35.905 times that of patients with lower TFRC expression, with a confidence interval from 4.859 to 265.3, and this result is statistically significant with a *p* value of 0.00045. (*** = *p* < 0.001).

**Table 6:** Multinomial Logistic Regression models: ODDS Ratios and significance.

	ODDs Ratios (*p* Values) Negative (Baseline)—Low/Medium Expressions; Negative (Baseline)—High Expressions								
Categorical Predictive Variable	TFRC	ACSL-4	ALOX-5	GPX4	Bmal 1	CLOCK	PER1	PER2	KLOTHO
Chronic Pathologies	0.19998 (0.27786); 0.03333 (0.01707 *)	1.938922 × 10^−4^ (0.80086); 3.115988 × 10^−5^ (0.75951)	-	0.11110 (0.1304533699); 0.03845515 (0.0224851804 *)	2.7779895 (0.4860337255); 16.6677060 (0.0398252071 *)	0.002197015 (0.8711087569); 18.752388604 (0.0214139418 *)	-	5.993170 × 10^3^ (0.822265475); 1.648554 × 10^4^ (0.802007556)	2.3999745 (0.549693368); 15.999163 (0.04288986 *)
Sex * Chronic Pathologies	-	Men: 2.901569 × 10^4^ (0); 1.140266 × 10^−4^ (0) Women: 3.121689 × 10^−9^ (0.8718058); 6.353824 × 10^−6^ (0.8954096)	-	-	-	Men: 1.664729 × 10^−1^ (0.952734092); 1.102400 × 10^7^ (0.988140529) Women: 1.969245 × 10^−5^ (0.984335245); 4.496620 × 10^0^ (0.32213980)	-	-	-
Smoking * Chronic Pathologies	6.419382 × 10^−2^ (0.9986503); 1.005612 × 10^2^ (0.9977298)	9.910429 × 10^−3^ (0.9744056); 2.752181 × 10^2^ (0.9689872)	1.091224× 10^−1^ (0.9529624); 6.847241 × 10^1^ (0.9104879)	1.481620 × 10^−2^ (0.9756463); 4.857345 × 10^2^ (0.9643524)	-	1.105501 × 10^−5^ (0.97687892); 8.126880 × 10^−5^ (0.98044592)	-	5.919619 × 10^3^ (0.89471417); 3.298729 × 10^−1^ (0.99769895)	1.275098 × 10^−3^ (0.90531676); 1.600727 × 10^−1^ (0.99842520)
Drinking * Chronic Pathologies	-	-	-	-	-	Low/Medium: 1 (1) High: no patients meeting both criteria	-	-	-
Obesity * Chronic Pathologies	-	5.392904 × 10^3^ (0.9938543); 3.649333 × 10^−4^ (0.9942142)	7.475973 × 10^−5^ (0.8966895049); 1.497775 × 10^−6^ (0.9501745593)	-	-	-	-	-	-
Diabesity * Chronic Pathologies	2.274799 × 10^4^ (0); 3.630096 × 10^−4^ (0)	-	-	1.046354 × 10^5^ (0); 9.790505 × 10^−5^ (0)	-	-	-	7.629219 × 10^4^ (0.935126805); 1.744353 × 10^−1^ (0.998204680)	-
Chronic Pathologies * Prior Malignancies	-	No patients meeting both criteria	-	-	-	No patients meeting both criteria	-	-	-

For each cell, there are two results representing the probability or ODDs ratio of transitioning from negative level of expression to low/medium and the probability or ODDs ratio of transitioning from low/medium to high. The numerical value for *p* values for each ODDs ratio are detailed in brackets. *p* value significance: * *p* < 0.05.

**Table 7:** Evaluation of numerical clinical variables with each protein marker’s levels of expression.

	Kruskal–Wallis *p* Value								
Numerical Predictive Parameter	TFRC	ACSL-4	ALOX-5	GPX4	Bmal 1	CLOCK	PER1	PER2	KLOTHO
Demographic									
Age	0.3644	0.471	0.3644	0.4166	0.1103	0.03833 *	0.1662	0.1885	0.8323
Carcinogenic marker									
Ca 19-9 U/mL (0–37)	0.287	0.2204	0.5617	0.02961 *	0.009228 *	0.07914	0.0473 *	0.002584 **	0.1434
CEA ng/mL (0–5)	0.3795	0.8859	0.5211	0.6901	0.1262	0.1561	0.5335	0.7981	0.4656
AFP ng/mL (0–13.4)	0.05113	0.5327	0.2548	0.1906	0.6212	0.3892	0.8572	0.7439	0.1147

*p* value significance: * *p* < 0.05, ** *p* < 0.01.

**Table 8:** Ordinal Logistic Regression (OLR) models: ODDs ratios and *p* values.

	ODDs Ratios (*p* Values) Negative (Baseline)—Low/Medium Expressions; Negative (Baseline)—High Expressions								
Numerical Predictive Parameter	TFRC	ACSL-4	ALOX-5	GPX4	Bmal 1	CLOCK	PER1	PER2	KLOTHO
Demographic									
Age	-	-	-	-	-	0.02106011 (0.1713099); 0.06174080 (0.3204356)	-	-	-
Carcinogenic marker									
Ca 19-9 U/mL (0–37)	-	-	-	0.2198436 (0.00839007 **); 1.3118077 (0.53830348)	0.5728256 (0.23735298); 3.1563382 (0.03480838)	-	0.6428608 (0.223576749); 2.2852821 (0.038850205 *)	0.3525962 (0.02491916 *); 2.6743104 (0.04756291 *)	-

For each cell, there are two results representing the probability or ODDs ratio of transitioning from negative level of expression to low/medium and the probability or ODDs ratio of transitioning from low/medium to high. The numerical values for *p* values for each ODDs are detailed in brackets. *p* value significance: * *p* < 0.05, ** *p* < 0.01.

## Data Availability

The datasets used and/or analyzed during the present study are available from the corresponding author upon reasonable request.

## Supplementary Materials

The following supporting information can be downloaded at: https://www.mdpi.com/article/10.3390/biom14080947/s1, SM1: Log-Rang Test for all the proteins studied; SM2: List of adjusted p values associated to Spearman coefficients from heatmap pairwise matrix; SM3: Fisher’s Exact Test; SM4: Adjusted p values for Fisher´s Exact Test; SM5: Kaplan Meier Survival according to the presence of a Chronic Pathology.
