# Supplementary material for: Abnormal Histopathological Expression of Klotho, Ferroptosis, and Circadian Clock Regulators in Pancreatic Ductal Adenocarcinoma: Prognostic Implications and Correlation Analyses
Source: Biomolecules. 2024 Aug 5;14(8):947. doi: 10.3390/biom14080947 (PMC11353028; doi:10.3390/biom14080947)

Supplementary Material

SM1. Log-Rang Test for all the proteins studied.

Protein marker	Chi-Square Statistic	Degrees of Freedom	P value
Ferroptosis markers			
**TFRC**	29.4	2	4e-07
**ACSL-4**	17.9	2	1e-04
**ALOX-5**	17.6	2	2e-04
**GPX4**	25.5	2	3e-06
Chronobiological markers			
**Bmal 1**	32.8	2	8e-08
**CLOCK**	41.3	2	1e-09
**PER1**	34.2	2	4e-08
**PER2**	28.5	2	6e-07
Antiaging markers			
**KLOTHO**	26.8	2	1e-06

SM2. List of adjusted p values associated to Spearman coefficients from heatmap pairwise matrix.

$\`TFRC-ACSL-4\`
[1] 2.088403e-06

$\`TFRC-ALOX-5\`
[1] 8.715189e-07

$\`TFRC-GPX4\`
[1] 2.922983e-08

$\`TFRC-Bmal 1\`
[1] 2.486562e-10

$\`TFRC-CLOCK\`
[1] 1.675504e-11

$\`TFRC-PER1\`
[1] 1.68284e-08

$\`TFRC-PER2\`
[1] 5.800521e-08

$\`TFRC-KLOTHO\`
[1] 1.74642e-08

$\`ACSL-4-ALOX-5\`
[1] 0.0295919

$\`ACSL-4-GPX4\`
[1] 2.24736e-05

$\`ACSL-4-Bmal 1\`
[1] 7.89156e-07

$\`ACSL-4-CLOCK\`
[1] 1.776848e-06

$\`ACSL-4-PER1\`
[1] 4.789733e-06

$\`ACSL-4-PER2\`
[1] 0.0006714387

$\`ACSL-4-KLOTHO\`
[1] 0.0004326312

$\`ALOX-5-GPX4\`
[1] 0.002685902

$\`ALOX-5-Bmal 1\`
[1] 0.002665569

$\`ALOX-5-CLOCK\`
[1] 5.715695e-05

$\`ALOX-5-PER1\`
[1] 1.059745e-05

$\`ALOX-5-PER2\`
[1] 0.0001228917

$\`ALOX-5-KLOTHO\`
[1] 9.621907e-05

$\`GPX4-Bmal 1\`
[1] 3.679174e-07

$\`GPX4-CLOCK\`
[1] 5.372383e-06

$\`GPX4-PER1\`
[1] 1.031502e-10

$\`GPX4-PER2\`
[1] 1.763828e-09

$\`GPX4-KLOTHO\`
[1] 0.0001177592

$\`Bmal 1-CLOCK\`
[1] 2.141798e-11

$\`Bmal 1-PER1\`
[1] 1.496232e-07

$\`Bmal 1-PER2\`
[1] 4.487139e-07

$\`Bmal 1-KLOTHO\`
[1] 1.473666e-06

$\`CLOCK-PER1\`
[1] 1.755143e-08

$\`CLOCK-PER2\`
[1] 2.698841e-07

$\`CLOCK-KLOTHO\`
[1] 1.664274e-05

$\`PER1-PER2\`
[1] 9.703349e-13

$\`PER1-KLOTHO\`
[1] 7.001588e-07

$\`PER2-KLOTHO\`
[1] 0.0001795237

SM3. Fisher's Exact Test.

Predictive Variable	Fisher's Exact Test (p-value)								
	TFRC	ACSL-4	ALOX-5	GPX4	Bmal 1	CLOCK	PER1	PER2	KLOTHO
Sex	1	0.5191	1	0.8851	1	0.1711	1	1	0.7074
Smoking Habit	0.8775	0.8449	0.5653	0.624	0.4223	0.1067	1	0.5658	0.4806
Drinking Habit	1	1	0.8469	0.8651	0.3479	0.4006	0.8087	0.7974	0.3106
Obesity	0.2061	0.5049	0.05488	0.2427	0.2866	0.35	0.2085	0.3695	0.1463
Type 2 Diabetes + Obesity (Diabesity)	0.8665	0.82	0.8665	0.4708	0.6351	0.5789	0.3551	0.08312	0.3251
Chronic Pathologies	0.02044 *	0.02064 *	0.06636	0.02695 *	0.04172 *	0.02246 *	0.05652	0.02567 *	0.0476 *
Prior Malignances	1	0.6015	0.8469	0.3655	0.4468	0.4006	0.4006	0.4006	0.5954
Sex * Smoking	1	0.7964	0.8527	0.9337	0.919	0.2969	0.9618	0.7703	0.8989
Sex * Drinking	1	0.9199	0.9797	0.9874	0.7981	0.3553	0.9935	1	0.5802
Sex * Obesity	0.4212	0.3534	0.08873	0.3961	0.4402	0.1514	0.3948	0.5767	0.1555
Sex * Diabesity	0.8471	0.2563	0.9794	0.09727	0.8131	0.3713	0.3988	0.1459	0.7326
Sex * Chronic Pathologies	0.06418	0.02608 *	0.2301	0.08922	0.2192	0.009979 **	0.2759	0.2048	0.1401
Sex * Prior Malignancies	1	0.7374	0.9797	0.8876	0.7981	0.3553	0.9935	0.92	0.9106
Smoking * Drinking	1	0.7154	0.8265	0.5959	0.7832	0.4194	0.5755	0.7046	0.1461
Smoking * Obesity	0.3121	0.5832	0.05585	0.265	0.4182	0.1411	0.3866	0.335	0.09417

Smoking * Diabesity	0.4866	0.9143	0.427	0.2952	0.7059	0.1847	0.2471	0.09622	0.1591
Smoking * Chronic Pathologies	0.01789 *	0.04123 *	0.04135 *	0.0265 *	0.1365	0.003577 **	0.7221	0.03784 *	0.009584 **
Smoking * Prior Malignancies	0.9724	0.5519	0.843	0.7595	0.4489	0.1457	0.9671	0.4929	0.6508
Drinking * Obesity	0.4239	0.8918	0.08958	0.4176	0.2978	0.405	0.5103	0.6414	0.09118
Drinking * Diabesity	0.9493	0.9418	0.8224	0.3901	0.7941	0.5919	0.4252	0.2002	0.2441
Drinking * Chronic Pathologies	0.0593	0.08467	0.1207	0.09436	0.1287	0.04726 *	0.1773	0.1218	0.04471 *
Drinking * Prior Malignancies	0.7313	0.8819	0.5278	0.6105	0.6616	0.6533	0.9949	0.9459	0.6084
Obesity * Chronic Pathologies	0.07219	0.04771 *	0.02615 *	0.07667	0.1251	0.06897	0.0521	0.05848	0.04224 *
Obesity * Prior Malignancies	0.4239	0.5704	0.08958	0.2347	0.24	0.405	0.5103	0.3743	0.173
Diabesity * Chronic Pathologies	0.008848 **	0.07057	0.05503	0.01582 *	0.06554	0.08929	0.15	0.01783 *	0.01337 *
Diabesity * Prior Malignancies	0.8593	0.7958	0.5118	0.4524	0.4534	0.5597	0.6375	0.0593	0.2399
Chronic Pathologies * Prior	0.0593	0.03953 *	0.1207	0.05765	0.1037	0.04726 *	0.1773	0.07268	0.09615

Malignancies									

P value significance: *<0.05, **<0.01, ***<0.001.

SM4. Adjusted p values for Fisher´s Exact Test

	Adjusted p-values (FDR)								
Predictive Variable	TFRC	ACSL-4	ALOX-5	GPX4	Bmal 1	CLOCK	PER1	PER2	KLOTHO
Chronic Pathologies	0.04312*	0.04312 *	-	0.04312 *	0.04771 *	0.04312 *	-	0.04312 *	0.04771 *
Sex * Chronic Pathologies	-	0.04312 *	-	-	-	0.04312 *	-	-	-
Smoking * Chronic Pathologies	0.04312 *	0.04771 *	0.04771 *	0.04312 *	-	0.04312 *	-	0.04771 *	0.04312 *
Drinking * Chronic Pathologies	-	-	-	-	-	0.04771 *	-	-	-
Obesity * Chronic Pathologies	-	0.04771 *	0.04312 *	-	-	-	-	-	-
Diabesity * Chronic Pathologies	0.04312 *	-	-	0.04312 *	-	-	-	0.04312 *	-
Chronic Pathologies * Prior Malignancies	-	0.04312 *	-	-	-	0.04312 *	-	-	-

False Discovery Rate correction (FDR) of the method Benjamini-Hochberg: adjusted alpha (0.001***; 0.01**; 0.05*): **0.003***; 0.015**; 0.05***.

SM5. Kaplan Meier Survival according to the presence of a Chronic Pathology

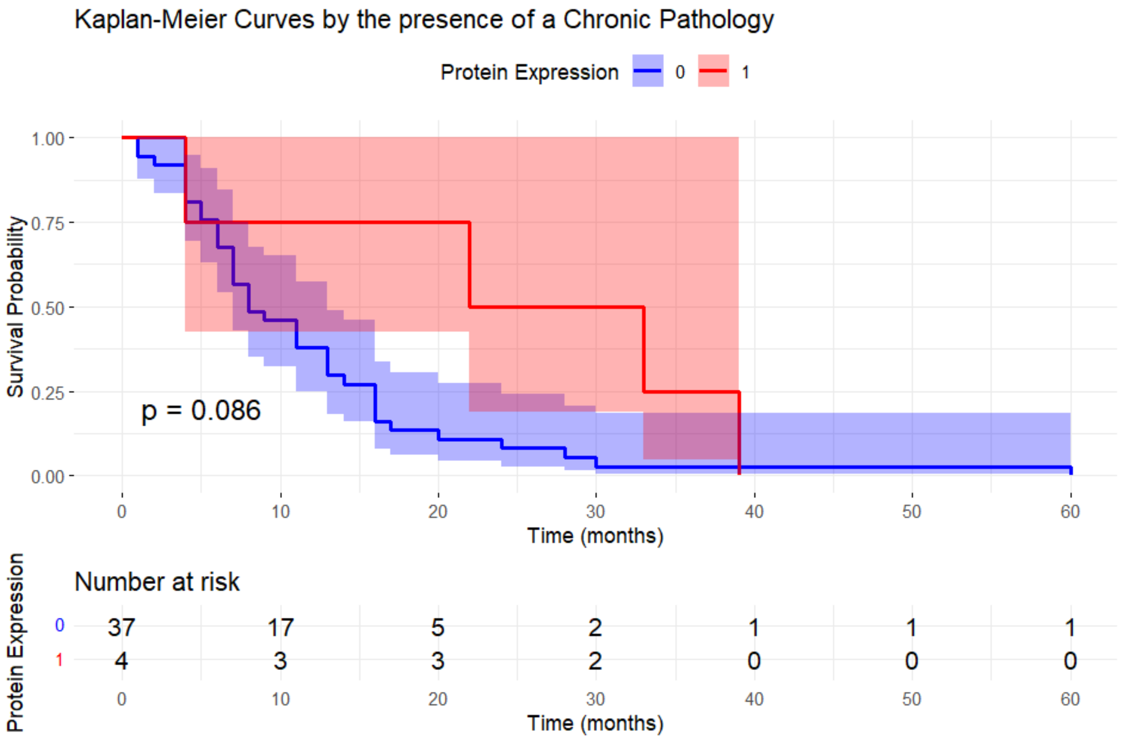

Supplement: Supplementary file 1 [file biomolecules-14-00947-s001.zip › biomolecules-3119243-supplementary.pdf]
